# Special Issue “Cellular and Molecular Biology of Cardiac Hypertrophy and Heart Failure: Pathogenesis, Diagnostics and Treatment”

**DOI:** 10.3390/ijms26146865

**Published:** 2025-07-17

**Authors:** Morris Karmazyn

**Affiliations:** Department of Physiology and Pharmacology, University of Western Ontario, London, ON N6A 5C1, Canada; morriskarmazyn@gmail.com

Heart failure represents a major medical concern of the 21st century. Currently, it is estimated that over 60 million individuals world-wide have heart failure. While the causes of heart failure are multifaceted, myocardial remodeling, including the development of maladaptive or pathological hypertrophy, represents a major contributor to the development of heart failure. Research into the mechanisms and therefore possible treatment targets associated with pathological hypertrophy has been extensive. This is particularly the case with respect to the challenging task of identifying the cellular and molecular processes underlying the myocardial hypertrophic process. Indeed, there has been substantial progress in identifying the exceedingly complex cellular and molecular mechanisms which contribute to myocardial remodeling and cardiomyocyte hypertrophy specifically [1]. While such advances have furthered our understanding of the hypertrophic program, they have at the same time revealed the complexity of this system with substantial underlying redundancy of these processes. Currently, some pharmacotherapy for treating heart failure does involve various medications which do indeed attenuate hypertrophy, such as angiotensin-converting enzyme inhibitors or angiotensin receptor blockers, which blunt the angiotensin 2-induced activation of the AT1 receptor that results in a pro-hypertrophic cell signaling cascade. It is clear, however, that new and effective approaches are needed. Nonetheless, there are numerous challenges facing investigators in delineating the key mechanisms underlying the hypertrophic program, particularly in view of the rapidly evolving progress in this field and the complexity underlying myocardial hypertrophy and heart failure processes. This Special Issue offers new directions as enunciated in its eight published articles, comprising animal and clinical studies as well review articles. These articles, which are briefly summarized below, can be found via the following link: https://www.mdpi.com/journal/ijms/special_issues/HJJZ6ZV053 (accessed on 5 July 2025).

An important component associated with myocardial remodeling and heart failure is defective energy metabolism, including mitochondrial dysfunction. Jia et al. show that aerobic exercise in mice subjected to hypertrophy and heart failure produced by thoracic aortic banding resulted in a reduction in the hypertrophic response and improved left ventricular function. In this study, the authors convincingly demonstrated that these beneficial effects were associated with diminished production of mitochondrial-derived reactive oxygen species resulting in improved mitochondrial function as well as a decrease in the degree of apoptosis. Overall, the study proposes the important concept of targeting mitochondrial dysfunction as a therapeutic approach for the treatment of heart failure.

Königstein and colleagues studied the potential role of the L-type Cav1.2 calcium channel in cardiac dysfunction associated with defective calcium homeostasis, a potential contributor to cardiac dysfunction associated with heart failure. Cav1.2 is encoded by the *Cacna1c* gene and the authors used *Cacna1c* haploinsufficient rats to demonstrate alterations in calcium handling of particular relevance to sympathetic stress of Cav1.2 and of potential broader significance to altered calcium handling in heart failure. These findings should advance future research into the mechanisms underlying defective calcium homeostasis associated with heart failure.

In their study, Weldrick and coauthors focus on the mechanisms which regulate the transition from the fetal heart gene program to the adult heart gene hypertrophic program with an emphasis on microRNAs as key factors in this process. Using mouse hearts collected at various times after birth, they clearly identified microRNA205, whose expression levels uniquely corresponded to the transition point. Additional studies in this report using various experiment approaches clearly implicate microRNA205 as a key regulator in the transition of cardiomyocytes from hyperplastic to hypertrophic growth. These findings advance our knowledge of cardiac development with respect to transition from hyperplastic to hypertrophic growth and serve as a novel basis for the treatment of cardiac hypertrophy preceding the development of heart failure.

Heger and coauthors aimed to assess the role of the transcriptional factor Y-box binding protein−1 (YB-1) as a negative regulator of the hypertrophic process. In initiating hypertrophy produced by phenylephrine-induced α1-adrenergic receptor activation using the H9c2 rat myoblast cell line and adult rat ventricular myocytes, the authors showed that suppression of YB-1 using interfering RNAs enhanced the hypertrophic response to phenylephrine upregulation of YB-1, while adenovirus vector infection prevented the hypertrophic phenotype, an effect specific for α1 receptor-induced hypertrophy. Moreover, the authors demonstrate significantly reduced YB-1 expression in cardiac biopsy samples from patients with end-stage heart failure. As the authors note, these findings are of substantial interest as they point to targeting YB-1 for the inhibition of pathological hypertrophy.

Liang and colleagues discuss the importance of histidine-containing dipeptides such as carnosine in controlling the levels of reactive carbonyl species, which are important contributors to myocardial remodeling. Analyzing plasma from patients undergoing cardiac surgery, the authors show that patients with left ventricular dysfunction have lower carnosine degradation rates than those with normal left ventricular function, suggesting that heart failure patients may benefit from oral carnosine supplementation.

A case report by Gencheva and coauthors demonstrated the importance of advanced diagnostic testing to identify unexplained hypertrophic cardiomyopathy. They report the case of a 48-year-old man with a history of atrial fibrillation and other vascular conditions including hypertension and stroke who developed hypertrophic cardiomyopathy and heart failure, which were associated with various gene abnormalities. The authors argue for a multifaceted use of imaging and molecular genetic analysis to identify individuals, particularly those at an active age, who are potentially at risk for developing hypertrophic cardiomyopathy and heart failure.

Two review articles are presented in this Special Issue. Dhalla and colleagues comprehensively review the literature related to right ventricular responses following myocardial infarction. They discuss the early adaptive response which evolves to subsequent maladaptive right ventricle hypertrophic responses following infarction. These right ventricle changes involve alterations in intracellular calcium handling and sympathetic adrenergic responses. The authors propose that right ventricular hypertrophy represents a compensatory mechanism in an attempt to maintain cardiac function in heart failure.

Adipocytes are now recognized as important contributors to physiology and pathophysiology by virtue of their ability to produce bioactive factors known as adipokines. Karmazyn and Gan review the potential role of one of the principal adipokines, leptin, in the hypertrophic process and review the cellular and molecular mechanisms underlying these effects. In addition to adipocytes, cardiac cells can also produce leptin and express leptin receptors. The review highlights the potential importance of leptin in heart failure, particularly under conditions of obesity, and suggests that targeting the leptin system may offer benefit for treating myocardial remodeling and heart failure.

This Special Issue highlights the complexity of the myocardial remodeling and heart failure processes, which involve a plethora of cellular and molecular mechanisms, some of which are raised in this issue. The future challenge is to apply our vast knowledge to therapeutic benefit. This is a difficult challenge in view of the underlying complexity but one which can likely be achieved through concerted integrative efforts. Another important, and indeed critical, area of research that has major clinical implication is to identify treatments that can not only attenuate but also reverse the myocardial remodeling and heart failure processes, a phenomenon which can be initiated via biological or physical approaches [2]. It is hoped that future studies will examine this in greater detail.
